# Accuracy and precision of non-invasive thermometers compared with the pulmonary artery temperature: a cross-sectional study

**DOI:** 10.1590/1516-3180.2023.0409.R1.05062024

**Published:** 2024-11-22

**Authors:** Rafael Lima Rodrigues de Carvalho, Mariana Avendanha Victoriano, Camila Claúdia Campos, Paula Frizera Vassallo, Vandack Nobre, Flávia Falci Ercole

**Affiliations:** I Adjunct Professor, Nursing School, Universidade Federal da Bahia (UFBA), Salvador (BA), Brazil.; II Education Manager, Proz Educação, São Paulo (SP), Brazil.; III Professor, Faculdade Anhanguera, Belo Horizonte (MG), Brazil.; IV General Intensive Therapy, Hospital das Clínicas, Universidade Federal de Minas Gerais (UFMG), Belo Horizonte (MG), Brazil.; V Titular Professor, Medicine School, Universidade Federal de Minas Gerais (UFMG), Belo Horizonte (MG), Brazil.; VI Titular Professor, School of Nursing, Universidade Federal de Minas Gerais (UFMG), Belo Horizonte (MG), Brazil.

**Keywords:** Thermometers, Fever, Temporal Arteries, Axillary, Tympanic, Oral and Pulmonary Artery

## Abstract

**BACKGROUND:**

Temperature fluctuations are critical indicators of a patient’s condition in intensive care units (ICUs). While invasive methods offer a more reliable measurement of core temperature, they carry greater risks of complications, limiting their use in most situations. This underscores the need for research evaluating the reliability of non-invasive temperature monitoring methods.

**OBJECTIVES:**

This study aimed to assess the accuracy and precision of four non-invasive temperature measurement techniques compared to pulmonary artery temperature, considered the gold standard.

**DESIGN AND SETTING:**

We conducted a cross-sectional clinical study with repeated measures in the ICUs at Hospital das Clínicas da Universidade Federal de Minas Gerais and Hospital Felício Rocho, Belo Horizonte, Brazil.

**METHODS:**

All patients admitted with a pulmonary artery catheter were included. We simultaneously recorded temperatures from the pulmonary artery, axillary area, oral cavity, temporal artery, and tympanic membrane. Bland-Altman plots were employed to assess the agreement between the different temperature measurements.

**RESULTS:**

A total of 48 patients participated, with a mean age of 54 years. Females comprised 66.67% of the sample. Compared to pulmonary artery temperature, the accuracy and precision (mean and standard deviation) of the non-invasive methods were: axillary (-0.42°C, 0.59°C), oral (-0.30°C, 0.37°C), tympanic membrane (-0.21°C, 0.44°C), and temporal artery (-0.25°C, 0.61°C). Notably, in patients with abnormal body temperature (non-normothermic), only oral and tympanic membrane methods maintained their accuracy and precision.

**CONCLUSIONS:**

The non-invasive thermometers evaluated in this study demonstrated acceptable accuracy and precision (within the clinically relevant threshold of 0.5°C) compared to pulmonary artery temperature. Among the non-invasive methods, the tympanic membrane measurement proved to be the most reliable, followed by the oral method.

## INTRODUCTION

Temperature monitoring is a crucial tool for hospitalized patients, especially those in intensive care units (ICUs).^1^ Abnormalities in body temperature (BT) are a common clinical sign, alerting healthcare personnel to potential infectious and other conditions. Fever is the most frequent manifestation,^2^ while hypothermia can also indicate poor outcomes.^3^ Additionally, BT can be used therapeutically, such as controlled hypothermia after cardiac arrest.^4^

In adults, hyperthermia is defined as a BT of 38.0°C or higher.^2^ Fever is typically defined as 38.3°C or above, although this may vary depending on patient characteristics, institutional protocols, and the measurement method use.^2^

Early detection of fever allows for prompt antibiotic therapy in life-threatening infections, particularly for vulnerable or critically ill patients.^2^ Fever can also trigger broader diagnostic investigations, not just for infections but also for other possibilities.^2^

Invasive thermometers, like pulmonary artery (PA) and bladder catheters, offer reliable temperature monitoring.^1-2^ However, despite their accuracy, invasive methods carry increased complication risks, limiting their routine use.^5^

While the literature lacks a consensus on the reliability of non-invasive methods, these technologies have seen advancements in algorithms improving their accuracy and precision.^1,6-7^ New thermometers and technologies are constantly emerging, but studies evaluating them remain scarce.^2^

Nurses and nurse assistants need to understand the appropriate type of temperature measurement for each clinical setting and patient, along with the associated reliability. This knowledge can lead to better patient assessments, allowing healthcare providers to identify patients with abnormal temperatures and intervene promptly.

## OBJECTIVE

This study aimed to evaluate the accuracy and precision of four non-invasive thermometers (axillary [AT], oral [OT], tympanic membrane [TM], and temporal artery [TA]) compared to the gold standard of PA catheter measurements. We also investigated factors that might influence the accuracy of these non-invasive methods.

## METHODS

This cross-sectional clinical study with repeated measures was conducted in three ICUs across two general hospitals in the southeast region of Brazil. Both hospitals are referral centers for high-complexity patients and have a total of 914 beds (Hospital 1: 486 beds, Hospital 2: 428 beds). Hospital 1 has a 16-bed mixed ICU unit. Hospital 2 has a 50-bed ICU unit further divided into 20 beds dedicated to cardiac patients and 30 mixed beds (surgical and medical).

This study was approved by the ethics committees of the Universidade Federal de Minas Gerais (71553317.7.0000.5149) and Hospital Felicio Rocho (71553317.7.3001.5125). Written informed consent was obtained from all patients or their next of kin.

### Patients

From December 2017 to December 2018, all adult patients (aged 18 years or older) admitted consecutively to the participating ICUs were screened for eligibility. To be included, patients had to have a PA catheter inserted either upon ICU admission or immediately before, Patients were excluded if they had technical difficulties preventing one of the five temperature measurements or if their PA catheter was removed before the first measurement.

Temperature measurements were taken three times at two-hour intervals.

### Study procedures

Four non-invasive thermometers were used: AT, OT, TM, and TA. An Omron^®^ clinical thermometer (Tokyo, Japan) was used to measure AT. The probe was placed in direct contact with the patient’s axillary skin at a 45º angle, the arm was closed, and the temperature was recorded after the beep. For OT, an Omron^®^ clinical thermometer (Tokyo, Japan) was used. The probe was placed in the sublingual pocket until the beeped. TM temperature was obtained using a Braun Thermoscan^®^ PRO 6000 (Kronberg im Taunus, Germany). The probe tip was placed in the ear canal as instructed by the manufacturer, the button was pressed, and the temperature was recorded. Finally, TA temperature was measured using an Exergen TAT 5000^®^ (Watertown, USA) device. The thermometer was slid across the forehead in a straight line while the button was pressed to record the temperature. All measurements were performed by the lead researcher (RLRC), following the manufacturer’s instructions.

Non-invasive temperatures were measured on the same side of the body every two hours, for a total of three measurements per patient. The site was chosen based on the patient’s position and the presence of invasive devices (endotracheal tubes, intravenous lines, and monitors).

We collected demographic (sex and age) and clinical data from all participants, including body mass index (BMI), main diagnosis at admission, current use of medications that could interfere with BT (antipyretics, vasodilators, and sedatives), vasopressor or inotrope requirement, use of mechanical ventilation (MV), use of an oxygen catheter, diaphoresis at the time of temperature measurement, bath time, ingestion of liquids or solids, and presence of ear wax.

### Statistical analysis

To assess the accuracy and precision of the non-invasive thermometers compared to the PA temperature, we calculated the difference between each device’s reading and the PA temperature. The mean of these differences represents the bias between each non-invasive method and the PA temperature, which reflects the accuracy of the non-invasive measurement. The variance of these differences represents the precision of the non-invasive temperatures, expressed as the standard deviation of the differences. Furthermore, Bland-Altman graphs were constructed for each thermometer to visually evaluate their accuracy and precision compared to the PA temperature.

To identify factors influencing the accuracy of each non-invasive method, we built linear regression models. These models included variables with a p-value < 0.20 in the univariate analysis. The four final models, one for each non-invasive temperature method, were calculated using the stepwise backward method. Post-hoc tests were performed to verify the model adjustment. We set a significance level of P < 0.05 for all statistical analyses.

## RESULTS

**Figure 1:**
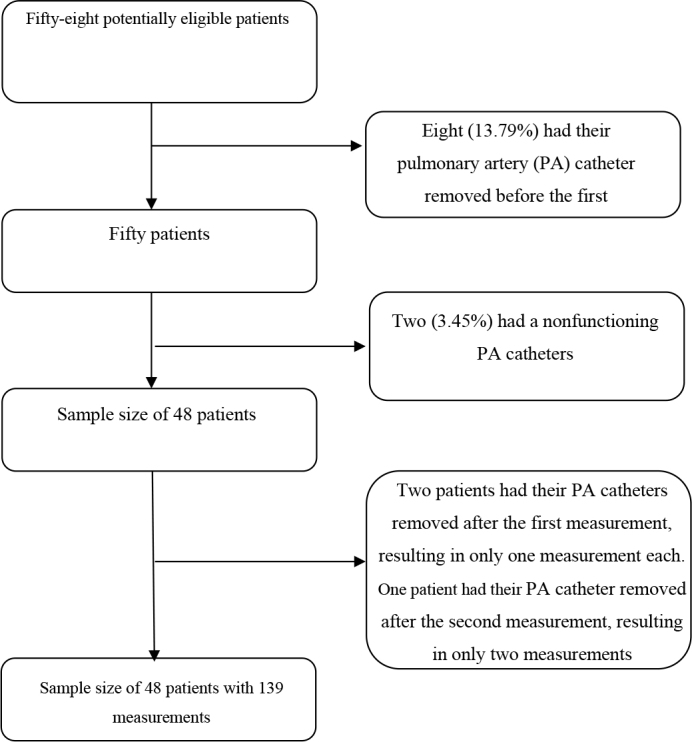
Flowchart of selection criteria and sample.

Fifty-eight patients were assessed for eligibility, of whom ten were excluded (**Figure 1**). Therefore, 48 participants were included in the final analysis, with 139 temperature measurements (mean of 2.9 measurements per patient).

Of the 48 patients, 15 were admitted to Hospital 1 and 33 to Hospital 2. Most patients were male (66.67%) with a mean age of 54 years (standard deviation ± 12.9). The primary characteristics of the 48 patients included in this study are presented in **Table 1**.

**Table 1:** Demographic and admittance data of 48 patients included in the study. Brazil, 2023

		n	%	Mean	Median	SD	IQR
**Sex**	Male	16	33,34	-	-	-	-
	Female	32	66,67	-	-	-	-
**Age**		-	-	54,36	56	12,96	50 – 62
**Height (meters)**		-	-	1.66	1.70	0.07	1.63 – 1.75
**Weight (kg)**		-	-	76.08	75	15.79	65 – 85
**BMI**		-	-	26.57	24.97	5.10	22.84 – 29.76
**Hospital**	1	15	31.25	-	-	-	-
	2	33	68.75	-	-	-	-
**Diagnoses**	Cirrhosis	31	64.58	-	-	-	-
	Other hepatic diseases	6	12.50	-	-	-	-
	Cardiovascular diseases	5	10.42	-	-	-	-
	Other diseases	6	12.08	-	-	-	-
**Total**		48	100%				

Most temperature measurements were performed while patients were receiving vasopressors: noradrenaline in 70.5% (98/139) and vasopressin in 13.7% (19/139) of measurements. Similarly, in 100 (71.94%) of the 139 temperature measurement episodes, patients were under MV. Finally, in 60 (43.12%) episodes, patients received sedatives, mainly fentanyl (39.57%).

Antipyretics were used four hours prior to temperature measurement in 19 episodes (13.87%), whereas a recent bath (less than one hour before temperature measurement) was recorded in eight (5.8%).

**Figure 2:**
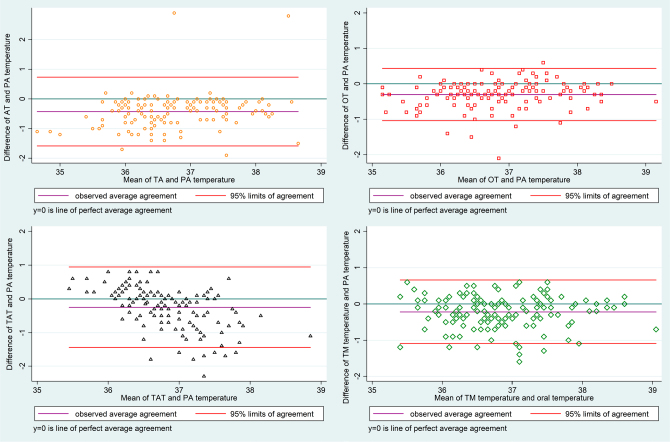
Bland-Altman plots of non-invasive temperature measurements compared to a pulmonary artery catheter.

The mean temperature obtained by the PA catheter across all measurements was 36.94°C (standard deviation ± 0.78). Among the non-invasive methods, TM showed the highest accuracy (-0.22°C), followed by TA (-0.25°C). OT had the best precision (0.38°C). Detailed data on temperature measurements and Bland-Altman plots are presented in **Table 2** and **Figure 2**, respectively.

**Table 2:** Temperature measurements, accuracy, and precision of 139 non-invasive measurements and pulmonary temperature. Brazil, 2023

Method	Mean	Interval (°C)	Accuracy	Precision	LOA
**Pulmonary artery**	36.94	35.2 – 39.4	-	-	-
**Axillary**	36.51	34.3 – 39.9	-0.427	0.592	-1.59 – 0.73
**Oral**	36.63	34.8 – 38.9	-0.303	0.376	-1.04 – 0.43
**Tympanic membrane**	36,72	34,8 – 38,7	-0.219	0.449	-1.10 – 0.66
**Temporal artery**	36,67	35,6 – 38,3	-0.250	0.95	-1.45 – 0.95

In the subgroup analysis restricted to abnormal temperature recordings (fever or hypothermia; n = 22), TM remained the most accurate (-0.17°C), followed by OT (-0.35°C). AT (0.41°C) and TA (-0.65°C) displayed lower accuracy. OT maintained the best precision (0.33°C), followed by TM (0.50°C). However, AT (0.90°C) and TA (0.99°C) showed greater bias compared to other methods.

We investigated factors potentially influencing the accuracy of each method compared to PA readings. The use of vasopressors, particularly nitroglycerin, negatively affected the accuracy of all four temperature measurement methods tested. Other vasoactive drugs like vasopressin and nitroprusside also impacted the accuracy of specific methods (OT and TA).

Interestingly, MV did not significantly alter the accuracy of OT (MV accuracy -0.300 vs non-MV accuracy -0.304, P = 0.95), and the presence of ear wax did not affect TM temperature accuracy (presence of ear wax accuracy -0.174 vs absence of ear wax accuracy -0.247, P = 0.350). **Table 3** provides a detailed breakdown of the identified factors influencing the accuracy of non-invasive thermometers compared to PA thermometers, as analyzed through multivariate linear regression **Table 3**.

**Table 3:** Factors that alter the accuracy of non-invasive measurements compared with pulmonary temperature after 139 measurements. Brazil 2023

Method	Factors that altered	Rate	P value
**Axillary**	BMI	0.02	0.038
	Bath before measurement	0.24	0.013
	Dose* of nitroglycerin	-0.02	< 0.001
**Oral**	Dose* of vasopressin	-0.01	0.008
	Dose* of nitroglycerin	-0.01	< 0.001
	Dose* of nitroprusside	-0.01	0.001
**Tympanic membrane**	Dose* of nitroglycerin	-0.01	< 0.001
**Temporal artery**	Age	0.01	0.02
	BMI	-0.04	< 0.001
	Dose* of vasopressin	-0.04	< 0.001
	Dose* of nitroglycerin	-0.02	< 0.001
	Dose* of nitroprusside	0.01	0.027

Dose is represented by the mg/hr of the drug infusion.

## DISCUSSION

This study compared the reliability of four non-invasive BT measurement methods to the gold standard, the PA catheter. Among the non-invasive methods, OT exhibited the greatest stability in patients with abnormal body temperature (not normothermic). Notably, vasopressor use emerged as the primary factor influencing the accuracy of non-invasive thermometers, affecting all methods tested.

Many studies have been conducted to assess the accuracy and precision of non-invasive thermometers.^8,9,10,11^ Most of these studies included a small sample of participants^8,12^ and lacked the analysis of factors influencing accuracy.^8,10^

While some prior studies reported divergent results, it is important to consider specific testing conditions. For example, one meta-analysis found poor agreement with TM thermometers in comparison to central thermometers.^1^ However, this finding may be specific to hypothermic patients, as other studies focusing on hypothermia also reported poor TM performance.^11,13^ In contrast, our study, which included a broader temperature range, identified TM as the most accurate non-invasive method. This aligns with other research highlighting the potential effectiveness of TM and OT for non-invasive temperature measurement.^6,8-9,14^

AT measurements showed mixed results, with good accuracy but poor precision compared to the PA catheter. This aligns with previous studies reporting similar findings.^1,2,5,9^

TA thermometers exhibited good accuracy (0.250°C) but lacked precision (0.950°C). This finding contributed to the ongoing discussion regarding TA reliability. While some studies advise against using TA in critical settings^1,2,5-6,15^ and question its effectiveness in identifying fever,^15^ others report its validity as a reliable method.^8,16^

This trend of good accuracy with poor precision for AT and TA contributed to the subgroup analysis of abnormal BTs (fever or hypothermia). While OT and TM remained the most reliable thermometers, AT and TA maintained good accuracy but lost precision.

The primary factor influencing temperature accuracy was the use of vasodilators (nitroglycerin and nitroprusside), affecting all four non-invasive methods. This aligns with previous research.^8-9^ This phenomenon likely stems from altered blood flow in the outer skin of patients receiving these medications, leading to discrepancies in temperature readings. Other factors impacting accuracy included BMI and recent baths (within an hour) for AT measurements, and BMI and age for TA measurements.

BMI can influence temperature measurements because a thicker layer of adipose tissue impedes heat conduction from deeper skin layers to the surface.^17^ Similarly, hot or cold baths before temperature measurement can alter skin blood flow and heat dissipation, potentially affecting AT readings.^18^ Age may play a role, as thinner skin in older adults allows for easier heat transfer from deep tissues to the outer skin.^19^

Our study identified TM and OT as the most reliable non-invasive methods. While OT is the preferred method for critically ill patients in the United States,^2,6^ it is less common in Brazil. Notably, the performance of both TM and OT remained relatively unaffected by fever or hypothermia. However, the small sample size of abnormal temperature measurements (22 of 139) limits definitive conclusions about their reliability in these specific conditions.

Our study employed a rigorous data collection methodology, utilizing advanced thermometers available in Brazil and including patients from two independent centers. One consideration for future research is to expand the sample size. While PA catheters are not routinely used in clinical practice, future studies might explore ways to recruit a larger patient population. Additionally, a larger sample size with a broader range of body temperatures, including more patients with fever or hypothermia, would allow for a more robust evaluation of accuracy and precision across diverse patient profiles.

## CONCLUSION

Our findings suggest that TM and OT are the most accurate and precise non-invasive methods compared to the gold-standard PA catheter. While AT and TA measurements fell within the clinically acceptable threshold, they exhibited lower precision. These data support the use of TM and OT for non-invasive temperature assessment in clinical practice. However, caution is advised when using non-invasive methods on patients receiving vasodilators or presenting with fever or hypothermia.
